# Research Progress on the Etiology and Pathogenesis of Alzheimer's Disease from the Perspective of Chronic Stress

**DOI:** 10.14336/AD.2022.1211

**Published:** 2023-08-01

**Authors:** Yun-sheng Liu, Hua-fu Zhao, Qian Li, Han-wei Cui, Guo-dong Huang

**Affiliations:** ^1^Department of Neurosurgery, Shenzhen Second People’s Hospital/the First Affiliated Hospital of Shenzhen University Health Science Center, Shenzhen, China.; ^2^The Central Laboratory, Shenzhen Second People’s Hospital/the First Affiliated Hospital of Shenzhen University Health Science Center, Shenzhen, China.; ^3^Central Laboratory, Shenzhen Samii Medical Center, Shenzhen, China.

**Keywords:** Chronic Stress, Alzheimer’s disease, Gut microbiota, Neuroinflammation, Glutamate system, Immune system

## Abstract

Due to its extremely complex pathogenesis, no effective drugs to prevent, delay progression, or cure Alzheimer’s disease (AD) exist at present. The main pathological features of AD are senile plaques composed of β-amyloid, neurofibrillary tangles formed by hyperphosphorylation of the tau protein, and degeneration or loss of neurons in the brain. Many risk factors associated with the onset of AD, including gene mutations, aging, traumatic brain injury, endocrine and cardiovascular diseases, education level, and obesity. Growing evidence points to chronic stress as one of the major risk factors for AD, as it can promote the onset and development of AD-related pathologies via a mechanism that is not well known. The use of murine stress models, including restraint, social isolation, noise, and unpredictable stress, has contributed to improving our understanding of the relationship between chronic stress and AD. This review summarizes the evidence derived from murine models on the pathological features associated with AD and the related molecular mechanisms induced by chronic stress. These results not only provide a retrospective interpretation for understanding the pathogenesis of AD, but also provide a window of opportunity for more effective preventive and identifying therapeutic strategies for stress-induced AD.

## 1. Introduction

Alzheimer’s disease (AD), a progressive degenerative disease of the central nervous system (CNS) characterized by learning and memory impairment and progressive decline in cognitive function, was first discovered and described by the German psychiatrist named Alois Alzheimer in 1906 [1]. With the gradual increase in human life expectancy, as well as improvements in the methods of diagnosis, the incidence and total number of AD cases are increasing every year. AD has become one of the most important threats to the quality of life and health of the elderly, posing a huge challenge to global economic development. The main pathological features of the AD brain are senile plaque depositions formed by the aggregation of β-amyloid (Aβ) and neurofibrillary tangles formed by hyperphosphorylation of the microtubule-associated protein tau, and neuronal loss in specific areas of the brain [2].

No drugs are available to cure AD. Current treatments approved by the US Food and Drug Administration are acetylcholinesterase inhibitors and memantine, a N-methyl-D-aspartate (NMDA) receptor antagonist. However, these drugs only improve the symptoms of AD and cannot reverse the course of the disease. The etiology and pathogenesis of AD are still unclear, and it is therefore difficult to find an accurate and effective treatment. Consequently, there is an urgent need to explore alternative strategies to prevent and treat AD.

**Figure 1.**
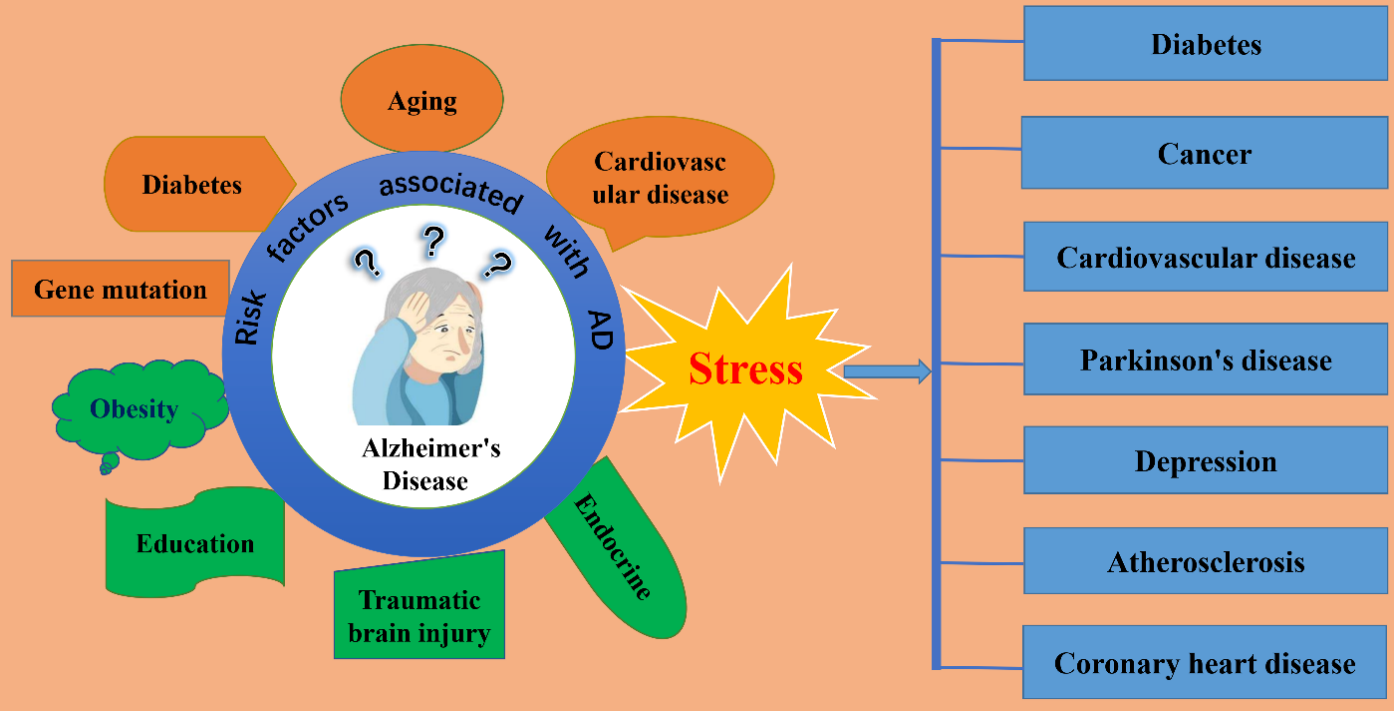
**Chronic stress is not only a risk factor for Alzheimer’s disease, but also for other age-related diseases**. The left side of the illustration depicts common factors known to influence the onset of Alzheimer’s disease, including stress, cardiovascular disease, aging, diabetes, gene mutation, obesity, education, traumatic brain injury, and endocrine function. The right-side lists age-related diseases for which stress is a risk factor, including diabetes, cancer, cardiovascular disease, Parkinson’s disease, depression, atherosclerosis, and coronary heart disease.

In addition to genetic mutations, there are many risk factors related to the onset of AD, including sex, aging, stress, traumatic brain injury, obesity, education level, and endocrine and cardiovascular factors [3, 4] (Fig. 1). Undoubtedly, advanced age is the most important factor. Stress includes both acute and chronic stress. Acute stress is generally believed to be the body’s adaptation to unfavorable environments or stimulation, and it always involves a protective response. However, in recent years, chronic stress has become an area of great interest in the study of AD etiology. Relevant studies have shown that environmental factors, especially exposure to chronic stress, can induce the onset of AD-related pathology in wild-type mice and worsen it in AD transgenic models [5-7]. Due to the complexity of the modern living environment and the variety of pressures we face daily, stress has become an inevitable component of everyday life. In-depth research on the mechanism by which chronic stress promotes the onset and development of AD-related pathologies could provide a theoretical basis for the development of effective clinical interventions.

To study the relationship between stress and AD, researchers have developed a large number of chronic stress animal models, including restraint stress, social isolation stress, noise stress, and unpredictable stress, which have been shown to induce AD-related symptoms. The development of these stress models provides a promising opportunity to study the relationship between stress and AD pathogenesis. However, the AD-like pathological features often differ between models, suggesting that the mechanisms involved may be different. This review summarizes the most common models for chronic stress currently used in research, describes the different AD-like pathological features induced by each of them, and explores the putative underlying mechanisms in order to provide some theoretical bases for the potential prevention and treatment of AD by regulating stress levels.

## 2. The history of stress

Stress is a syndrome caused by the body’s adaptive response to various internal and external environmental stimuli, as proposed by Hans Selye in 1936 [8]. In another words, stress describes the response to an experience that is emotionally and physiologically challenging [9]. It has been proposed that constant stress could dismantle biochemical protection mechanisms, thus making individuals vulnerable to attack by diseases. It is generally believed that the negative impact of excessive stress on health manifests in two aspects: one is to aggravate existing diseases by weakening protection mechanisms, and the other is to enhance the vulnerability of certain organs to diseases. Owing to excessive work pressure and a complex living environment, individuals are susceptible to continuous stress. In the 1970s, Mason proposed a theory of psychological stress to explain the mechanism underlying some mental illnesses. Plenty of clinical data shows that stress is closely related to the onset of many age-related diseases, including diabetes, cancer, cardiovascular disease, Parkinson's disease, depression, atherosclerosis, and coronary heart disease, as well as Alzheimer’s disease [10-14] (Fig. 1). As life rhythms accelerate and more stressful events need to be faced daily, the prevalence and number of diseases caused by stress, and their associated economic burden, are also increasing every year.

**Figure 2.**
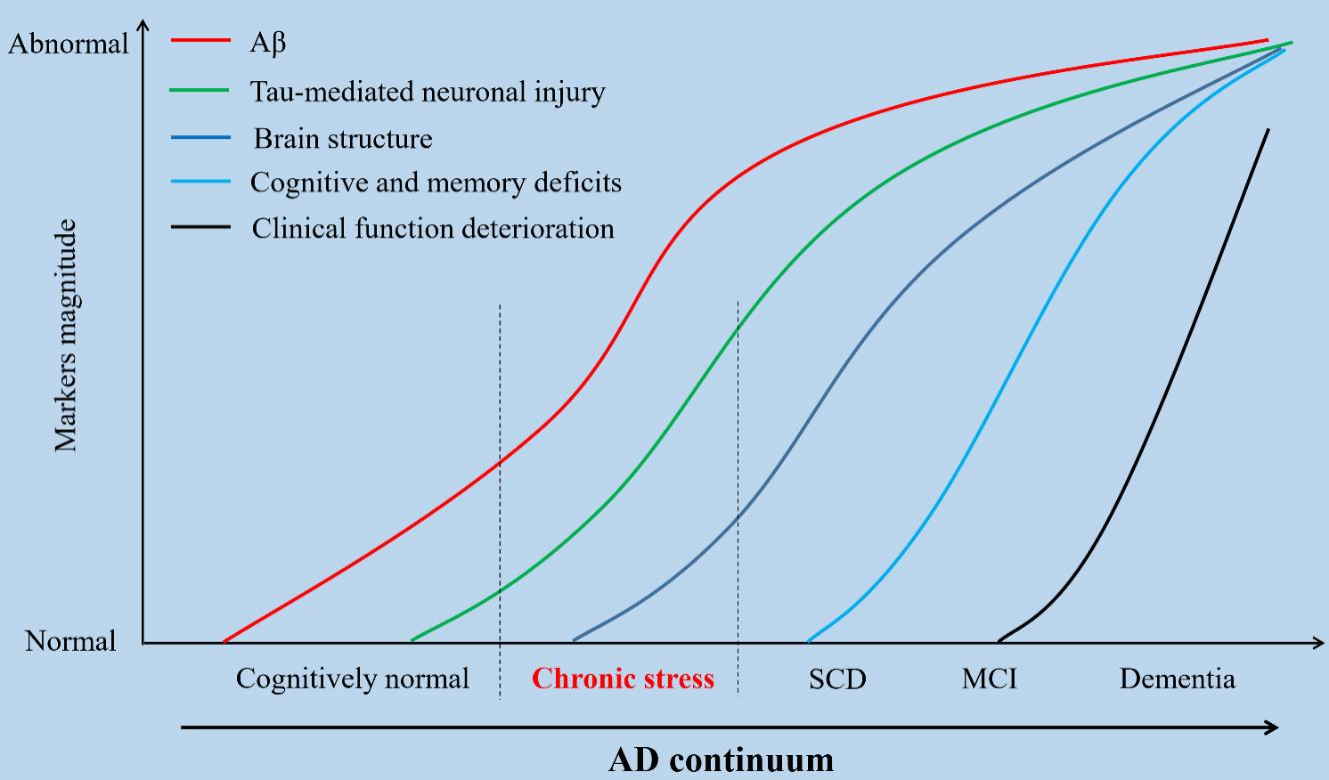
**Theoretical dynamics of the AD continuum**. Changes in dynamic biomarkers (Aβ, tau-mediated neuronal injury, brain structure, cognitive and memory deficits, clinical function deterioration) during the AD pathological cascade are shown. Chronic stress precedes and acts as a trigger for subjective cognitive decline (SCD), which may precede mild cognitive impairment (MCI), which in turn becomes manifests before the early clinical symptoms of dementia. Aβ, amyloid β.

The human brain is susceptible to stressful events, and an increasing number of studies on AD-specific neuropathological changes elicited by stress have recently attracted intense attention. Chronic stress may precede and trigger subjective cognitive decline (SCD), which precedes mild cognitive impairment (MCI), which in turn often occurs before the early clinical symptoms of AD [15] (Fig. 2). Chronic stress can change neuronal properties in the brain and disturb learning, memory and cognitive processes, suggesting that this event may function as a trigger for AD pathology.

However, due to the inherent difficulty of performing a large-scale study of the relationship between stress and AD pathogenesis in human subjects, choosing a suitable animal model is crucial. This review describes several models for chronic stress commonly used in current research, and the insight on the mechanisms by which stress promotes the onset and development of AD that has been gained from these models.

## 3. Chronic restraint stress

The chronic restraint stress (CRS) animal model is used as one of the techniques used to monitor physiological and psychological changes caused by stress. Under this model, the animals are repeatedly placed in conical tubes with holes for ventilation for half an hour to several hours per day during several weeks or months, without physical pressure or pain involved [16]. It has been reported that CRS can promote AD-type pathology in wild type rats and mice, and in transgenic models for AD, including aggregation and deposition of Aβ, hyper-phosphorylation of tau protein, and degeneration and massive loss of neurons, as well as decline in learning and memory ability (Table 1) [7, 17-20].

**Table 1:** Rodent studies on impact of chronic restraint stress (CRS) on AD-related markers.

Stress paradigm	Procedure	Animal model	Relevance to AD	Ref.
**CRS**	Mice were placed in 50 ml conical tubes with holes for ventilating, 30 min each day for 18 days.	C57BL/6 mouse	p-tau ↑ Tau insolubility ↑	6
	Mice were restrained in conical tubes for 6 h/d for 6 d/week, and for 4 weeks.	Tg2576 mouse	Aβ_40_, Aβ_42_ and p-tau ↑ Neurodegeneration	7
	Rats were placed in flat-bottom acrylic restrainers with ventilation for a daily episode of 30 min of restraint for 14 days.	Wistar rat	p-tau ↑	17
	The stress was performed with a stainless mesh that allowed for a close fit to rats for 6 h/d for 21days.	Fisher rat	Neuronal loss and cognitive dysfunction	18
	Mouse was subjected to 6 h/d of immobilization stress in a mouse stress box (4.5W×10L×4.5H cm), for 4 days per week, and for 8 months.	APP_V717I_-CT100 transgenic mouse	Learning and memory impairments Aβ and APP-CTFs ↑	19
	Rats were restrained in Plexiglas tubes for 6 h/d for 21 days.	Sprague Dawley rat	Impairment in memory performance and the loss of dendritic spines	20
	Mice were restrained in ventilated 50-mL conical tubes for 30 min/d for 14 days.	C57BL/6 mouse	p-tau ↑ tau filaments and aggregate ↑	25
	Mice were placed into well-ventilated 50-mL conical tubes for 6 h/d for 21 days.	C57BL/6 mouse	Learning and memory deficits	51
	Mice were placed head-first into a well-ventilated 50 ml polypropylene conical tubes for 2 h/d for 14 days.	B6.Cg-TgN transgenic mouse	Dendrite and dendritic spine loss	57
	Mice were placed in modified 50-mL centrifuge tubes for 1 h per day for 14 days.	C57BL/6 mouse	Cognitive Impairments	58

### 3.1 HPA axis

**Figure 3.**
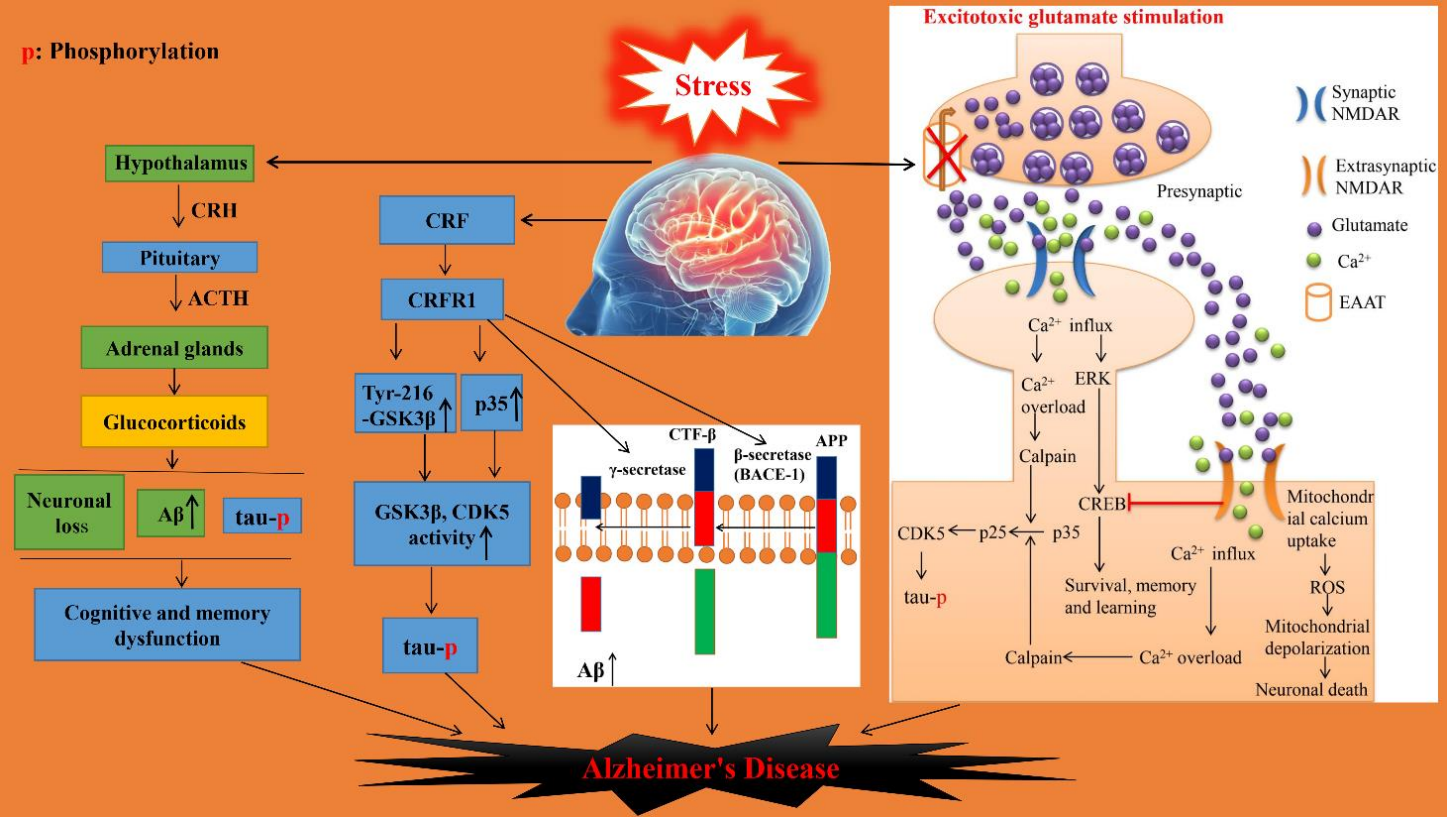
**Chronic restraint stress (CRS) induces AD pathogenesis by disrupting the HPA axis, and the CRF and glutamate signaling pathways**. (1) CRS induces an increase in circulating glucocorticoids through the HPA axis, which in turn promotes an increase in Aβ generation, tau phosphorylation, and neuronal degeneration. (2) CRS promotes tau phosphorylation and Aβ generation through the corticotropin-releasing factor (CRF) pathway. (3) CRS inhibits the expression of glutamate transporter EAAT, leading to persistent excitatory toxicity in neurons. On one hand, excitotoxicity induces tau protein phosphorylation through Ca^2+^ overload mediated by synaptic and extrasynaptic NMDA receptors (NMDARs). On the other hand, activated extrasynaptic NMDARs induce neuronal apoptosis by excess ROS production. Moreover, activated extrasynaptic NMDARs can also inhibit the CREB signaling pathway, which mediates the physiological effects of synaptic NMDARs.

Studies have found that the HPA (hypothalamic-pituitary-adrenal) axis is dysfunctional in AD, and the basal corticosterone level is significantly increased [21]. After stress stimulation, corticotrophin-releasing hormone (CRH) secreted by the hypothalamus acts on the pituitary gland to induce the release of adrenocorticotropic hormone (ACTH), which further acts on the adrenal gland to promote the release of glucocorticoids (corticosterone (CORT) in mice and rats, cortisol in primates) [22]. The increase in the level of CORT is associated with damage to synaptic function in AD (Fig. 3). Chronic stress or administration of CORT can accelerate the degradation of hippocampal function and cause AD-like lesions, including neuronal loss, increased Aβ deposition, tau phosphorylation, and loss of cognitive and memory function [23]. However, it has been reported that transgenic mice expressing a mutated form of tau (PS19) that were implanted with slow-release subcutaneous CORT pellets failed to show an increase in tau phosphorylation, suggesting that CORT may not be the key hormones mediating tau phosphorylation in response to stress [7].

### 3.2 CRF system

The expression of corticotropin-releasing factor (CRF) and its receptor, CRFR, is significantly increased in the brains of AD [24]. Studies have shown that CRF may play a crucial role in stress-induced Aβ increase and tau phosphorylation, which can accelerate the pathogenesis of AD (Fig. 3); These results were not only confirmed in wild type mice, but also in AD transgenic mice, which indicate that CRF plays a propelling role in the progression of AD [7, 25]. The CRF receptor is composed of two subtypes: CRFR1 and CRFR2. Rissman et al. found that CRFR2 can inhibit tau phosphorylation induced by stress [26]. However, most studies concluded that CRFR1 plays a positive regulatory role in the stress response [7, 27]. Tau phosphorylation and learning and memory impairment triggered by stress were reversed in AD transgenic mice receiving CRFR1 antagonist [7], CRFR1 knockout mice, and wild-type C57BL/6 mice treated with CRFR1 antagonist [25]. The mechanism involved was CRFR1 upregulation of the active form (Tyr-216 phosphorylated) of glycogen synthase kinase 3β (GSK3β) and alteration of p35 expression levels [26]. It is well known that GSK3β and cell cycle-dependent protein kinase 5 (CDK5) are the two most important kinases in the brain regulating tau protein phosphorylation, and CDK5 activity requires p35 or p25 regulatory subunits. Increased tau phosphorylation and Aβ levels lead to synaptic degeneration, which in turn impairs learning and memory behavior in mice [28].

Aβ is derived from the cleavage of the amyloid precursor protein (APP) by β- and γ-secretases [29]. β-secretase (BACE-1) cleaves APP to generate the C99 fragment (also known as APP CTF-β), which comprises the N-terminus of Aβ [30]. When γ-secretase cleaves CTF-β, Aβ is released in several forms of fragments, the most abundant of which is composed of Aβ_38_, Aβ_40_, and Aβ_42_, respectively. Aβ_40_ and Aβ_42_ peptide monomers are flexible hydrophobic peptides that can rapidly self-aggregate into pro-inflammatory dimers, oligomers, and fibrils to drive AD pathogenesis [31]. The removal and elimination of excessive Aβ_40_ and Aβ_42_ peptide monomers represents a promising strategy for preventing AD. APP CTF-β and Aβ peptides were significantly reduced in the brains of mice treated with CRFR1 antagonists, and in CRFR1 knockout mice [32, 33], indicating that CRFR1 is regulates β- or γ-secretase expression, activity, and/or trafficking (Fig. 3).

### 3.3 Glutamate system

Glutamate is the most important excitatory neurotransmitter with the highest content in the CNS. As a neurotransmitter, glutamate must be eliminated immediately after it is released into the synaptic cleft to prevent excitatory toxicity. Glutamate is eliminated mainly through reuptake, relying on the high-affinity excitatory amino acid transporters (EAAT) 1-5 expressed on the presynaptic and glial cell membranes [34]. Among them, EAAT1 is mainly present in the cerebellum, hippocampus, cortex, and striatum, whereas EAAT2 is mainly distributed to the cerebral cortex, hippocampus, and striatum. They both belong to the glial glutamate transporter family and bind glutamate with high affinity. Therefore, any reduction in EAAT1 and EAAT2 protein levels directly affects extracellular glutamate concentration. EAAT2 accounts for 95% of the uptake and transport of glutamate, and EAAT2 deficiency shows overlap with human aging and AD at the transcriptomic level [35]. Shan et al. found that after CRS, the expression of EAAT1 and EAAT2 in the brains of wild type mice decreased in both total protein and membrane-bound forms, and the decrease in the membrane components of the brains of aged mice was more remarkable than that in younger subjects [6].

NMDARs are glutamate-gated ion channels that are critical for neuronal communication and play a vital role in the dysfunction of glutamatergic transmission [36]. Our previous study found that CRS increased the expression levels of the glutamate NMDAR subunits GluN2A and GluN2B in the hippocampus and prefrontal cortex of wild type mice [37]. GluN2A and GluN2B can be detected in both synaptic and extrasynaptic NMDARs in the adult brain, with GluN2A mainly located in synaptic NMDARs and GluN2B mainly in extrasynaptic NMDARs [38]. In addition, increased expression of extrasynaptic GluN2B was found in the brain of APP/PS1 transgenic mice, and xanthoceraside significantly improved the learning and memory behavior in APP/PS1 transgenic mice by inhibiting the overexpression of extrasynaptic GluN2B [39]. Stimulation of NMDARs at synaptic sites with physiological concentrations of glutamate promotes cell survival, learning, and memory via the Ca^2+^-ERK-CREB pathway. However, prolonged activation of extrasynaptic NMDARs with excitotoxic glutamate stimulation causes calcium overload and neuronal apoptosis. The initial calcium influx following excitotoxic glutamate stimulation triggers a secondary intracellular calcium overload, and this secondary response strongly correlates with neuronal injury and death. The main reason is the involvement of mitochondria in the maintenance of cellular calcium homeostasis. Mitochondria can restore intracellular calcium concentrations by absorbing large amounts of calcium. In response to excitotoxic glutamate stimulation, the mitochondrial uptake of calcium results in the generation of an excessive amount of reactive oxygen species (ROS), leading to mitochondrial depolarization and excitotoxic neuronal death [40]. In addition, the activation of extrasynaptic NMDARs can also inhibit the ERK signaling pathway, which mediates the physiological function of synaptic NMDARs [38, 41].

Memantine is a voltage-dependent noncompetitive antagonist of NMDAR channels that preferentially binds to extrasynaptic NMDARs and blocks their hyperactivation without affecting the synaptic NMDAR signaling underlying various physiological functions [42, 43]. Compared with other ionotropic glutamate receptors, NMDARs have stronger permeability to Ca^2+^. Under normal circumstances, Ca^2+^ influx regulates physiological processes such as synaptic plasticity [44]. However, excessive intracellular Ca^2+^ levels can also cause excitotoxicity. Chronic stress induces the influx of large amounts of Ca^2+^ into neurons through synaptic and extrasynaptic NMDARs, and persistent Ca^2+^ overload in neurons leads to progressive dysregulation of synaptic function, and ultimately to neuronal cell death. This involves the activation of the Ca^2+^/calpain pathway by Ca^2+^ overload in neuronal cells, which in turn activates CDK5 through the Calpain/p25/CDK5 pathway to promote the phosphorylation of tau protein. This provides theoretical support for the clinical use of memantine in the prevention and treatment of stress-induced AD. The above results also suggest that abnormal glutamate signaling system is involved in the AD-related pathology induced by CRS (Fig. 3).

### 3.4 Neuroinflammation

**Figure 4.**
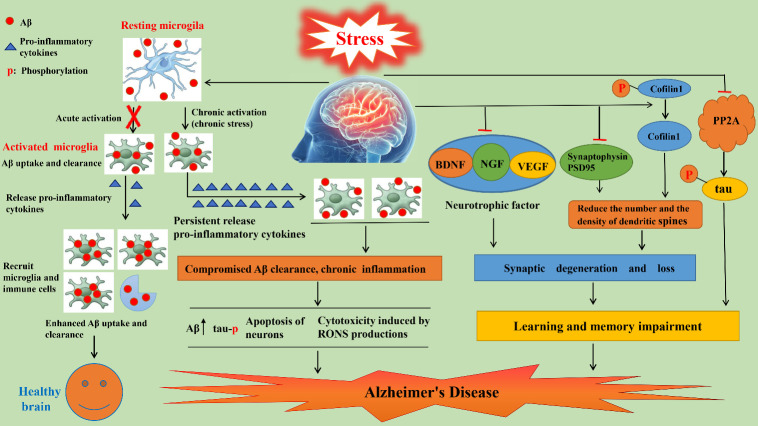
**The mechanism of CRS-induced AD pathogenesis**. (1) Under physiological conditions, neuroinflammation contributes to Aβ clearance, whereas a continuous and excessive inflammatory response induced by CRS compromises Aβ uptake by microglia. This promotes AD-related pathology, namely, an increase in Aβ production and tau phosphorylation. Ultimately, synaptic function is damaged and neuronal apoptosis is induced. (2) CRS inhibits the expression of neurotrophic factors (BDNF, VEGF, NGF) and synapse-associated proteins (PSD-95, synaptophysin), reduces the expression of PP2A, and promotes the dephosphorylation of cofilin1 at the Ser3 site, leading to degeneration and loss of synapses, which contribute to the onset of AD.

Neuroinflammation is a common pathological feature of AD. To reduce the damage caused by stress to the body, microglia and astrocytes secrete inflammatory factors that promote immune cells recruitment, thereby clearing infected or damaged tissues and cells. Microglia are the main immune cells in the brain and play a crucial role in the maintenance of brain homeostasis and immune surveillance [45]. When Aβ is abnormally deposited, microglia can engulf the aggregated proteins and secrete pro-inflammatory cytokines to recruit additional microglia and immune cells, enhancing Aβ uptake and clearance [46, 47]. However, although this initial immune response has a beneficial effect by removing accumulated Aβ, the persistent inflammatory response caused by CRS is harmful to neuronal survival and synaptic function, and eventually also damages the phagocytosis of Aβ by microglia [48]. Furthermore, the excessive release of inflammatory factors may even promote Aβ generation and tau hyperphosphorylation [49, 50]. Therefore, neuroinflammation plays a dual role in AD pathogenesis, under physiological conditions, it contributes to the clearance excess of Aβ secreted by nerve cells, while a continuous and excessive inflammatory response directly promotes AD-related pathology (Fig. 4). Increased inflammation is a common event in brains affected by chronic stress and may represent one mechanism by which stress promotes AD pathology.

### 3.5 Synaptic dysfunction

Synaptic dysfunction is closely related to learning and memory impairment. Studies have found that CRS can not only reduce the number and density of hippocampal dendritic spines but also reduce the expression levels of the presynaptic membrane marker synaptophysin, the postsynaptic membrane marker post-synaptic density-95 (PSD-95), and the neurotrophic factors, such as brain-derived neurotrophic factor (BDNF), vascular endothelial growth factor (VEGF), and nerve growth factor (NGF) [51-53] (Fig. 4). BDNF plays an important role in synaptogenesis and cognition by promoting synaptic plasticity, and synaptogenesis [54]. VEGF can improve cognition by reducing Aβ production [55]. In addition, studies have shown that a decline in serum VEGF levels is directly associated with the onset of AD [56]. Finally, NGF is essential for neuronal survival.

### 3.6 Cofilin1 and PP2A

Cofilin1 is essential for the growth and remodeling of dendrites and dendritic spines. Downregulation of cofilin activity can increase dendritic spine density. Cofilin1 is activated by dephosphorylation at Ser3, and CRS can promote the dephosphorylation at this particular site, increasing cofilin activity. This leads to the reduction in the number of dendritic spines and has an impact on learning and memory in mice [57] (Fig. 4). Zhang et al. showed that CRS inhibits the expression of the protease PP2A, which is a crucial enzyme for the dephosphorylation of tau. Approximately 70% of the phosphorylated tau is dephosphorylated by PP2A, and a reduction in PP2A expression leads to the aggregation of hyperphosphorylated tau and to a disruption in memory function in mice [58] (Fig. 4).

### 3.7 Microbiota-gut-brain axis

**Figure 5.**
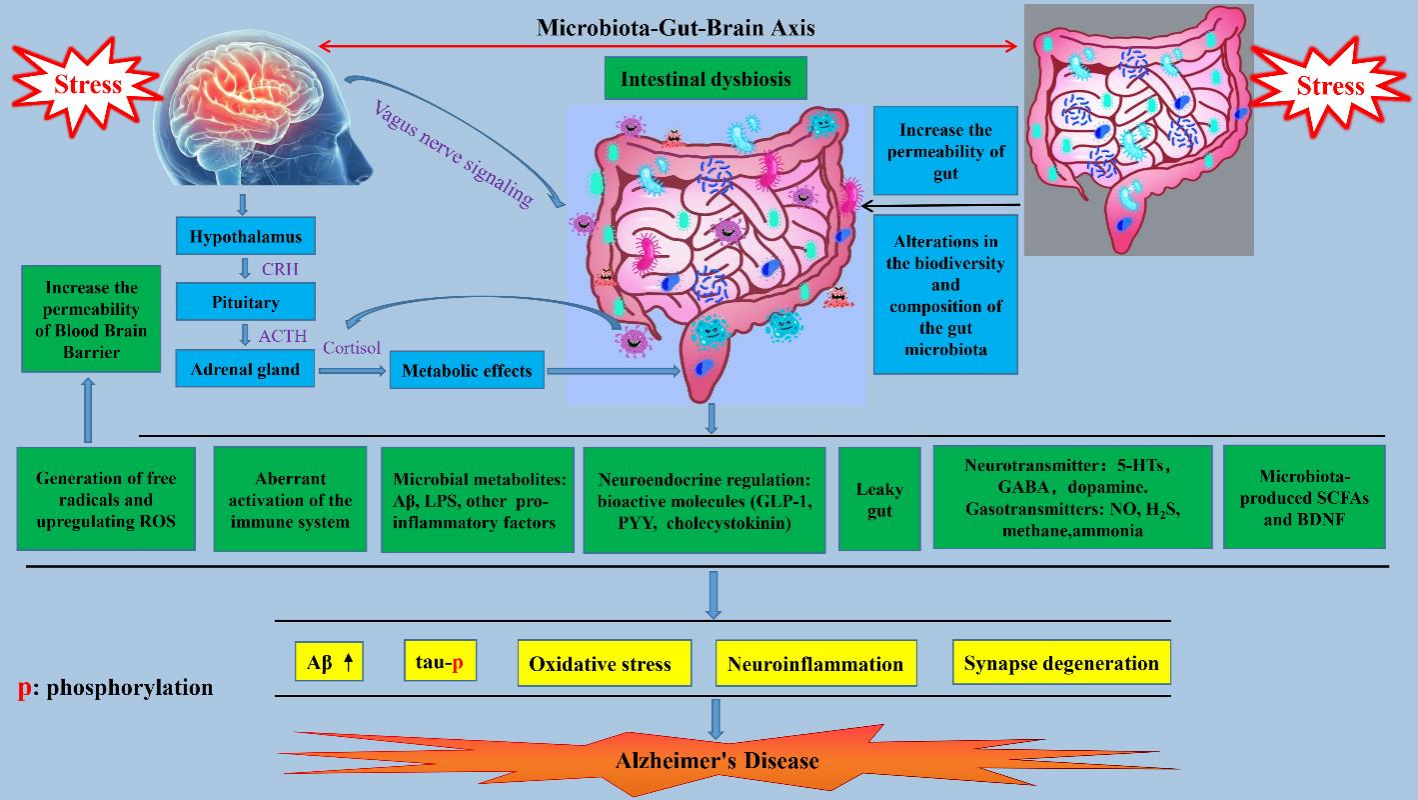
**Chronic restraint stress (CRS) induces AD pathogenesis by disrupting the gut microbiota**. Dysbiosis in the gut microbiota induced by CRS is linked to AD pathology in various ways, which include increased permeability of the gut, vasculature, and blood-brain barrier; increased amyloid burden; abnormal secretion of LPS; misregulation of the HPA axis and vagus nerve signaling; neuroinflammation; oxidative stress; aberrant immune activity; alterations in the biodiversity and composition of the gut microbiota; downregulation of BDNF and SCFAs; decreased secretion of related neurotransmitters; and abnormal release of gasotransmitters of microbial origin.

“All disease begins in the gut” was purportedly said more than 2,000 years ago by Hippocrates, a Greek physician known as the father of modern medicine [59]. The gut microbiota in humans contains approximately 10^14^ microbes that outnumber the host’s cells by approximately ten-to-one [60, 61]. The function of the gut microbiota was previously thought to be limited to maintaining normal gastrointestinal function, but they also regulate several additional processes, including vitamin and glucose metabolism, immune and inflammatory responses, central and peripheral neurotransmission [62, 63]. Growing evidence has indicated that the gut microbiota plays an active role in obesity, addiction, type 2 diabetes mellitus, cancer, aging, pain, stroke, and even neurodegenerative diseases [59]. In 2018, Lu et al. observed significant memory deficits in germ-free mice, suggesting an essential role of the gut microbiota in memory maintenance [64]. Indeed, numerous studies have provided evidence that *Lactobacillus* and *Bifidobacterium* probiotic supplements have a positive effect on mouse memory, assessed by object recognition and fear conditioning tests [65, 66]. Moreover, alterations in the biodiversity and composition of the intestinal microbiota have been observed in AD patients as well as in mouse models for AD [67, 68]. Liu et al. studied 97 participants from Hangzhou (China), including 32 healthy controls, 32 patients with amnestic mild cognitive impairment, and 33 patients with AD, and found marked differences in microbiota composition between the groups. Moreover, these alterations were tightly correlated with AD severity [69]. Kim found that transplantation of fecal microbiota from wild type mice mitigated amyloid and tau pathology, memory deficits, and reactive gliosis in an AD mouse model [70]. Probiotics (*Lactobacillus* and *Bifidobacterium*) ingestion by AD patients and murine AD models reduced various pathological markers, such as brain atrophy, Aβ accumulation, learning and memory deficits, and oxidative stress [71, 72]. More recently, mounting evidence has suggested that gut microbiota dysbiosis induced by CRS affects brain structure and function, and individual behavior through the microbiota-gut-brain axis, leading to the onset and development of AD [73, 74]. The microbiota-gut-brain axis is a bidirectional communication system comprising several routes, including the neural and immune systems, microbial metabolites, and endocrine signals (Fig. 5).

Here, we discuss the mechanisms underlying dysbiosis of the gut microbiota may participate in AD onset. It is known that CRS can accelerate aging, and Lee et al. found that transfer of gut microbiota from aged mice to younger mice was sufficient to reproduce the cognitive decline associated with aging [75]. Multiple reports have confirmed that the gut microbiota naturally secrete massive amounts of Aβ, lipopolysaccharide (LPS), and related microbial secretory products. Considering the huge number of microbes that comprise the human gut microbiota, it is apparent that we need to have an in-built tolerance to life-long exposure to LPS, Aβ and other related pro-inflammatory pathogenic signals. This exposure is nevertheless likely to increase the burden of amyloid protein and LPS in the CNS and activate the microglial-mediated innate immune and inflammatory responses. In individuals suffering from chronic stress, this may further contribute to AD development.

As mentioned for neuroinflammation, CRS can attenuate amyloid plaque sensing, phagocytosis, and clearance by microglia. Moreover, CRS is known to induce gut microbiota dysbiosis characterized by increased intestinal permeability and changes in gastrointestinal motility leading to “leaky gut”. As a result, bacteria, pathogens, amyloid protein and LPS can freely cross the epithelial barrier [66]. Extracellular Aβ deposition can cause secondary pathological changes such as tau hyperphosphorylation, oxidative stress, neuroinflammation, synaptic degeneration, and neuronal death, eventually leading to AD [76].

Gram-negative bacteria are predominant in the gut microbiota, and LPS is the major component of their cell wall. The secretory products of the gut microbiota are seriously powerful immune activators and pro-inflammatory factors that affect the host, accelerating the free radical’s production and upregulating ROS and/or reactive nitrogen species. This subsequently increases vascular and blood-brain barrier (BBB) permeability, immunogenicity, and aberrant activation of the immune system [77]. The increased permeability of the gut, vasculature, and BBB results in large amounts of Aβ protein and LPS leaking into the CNS and the peripheral circulation, which contributes to the accumulation of amyloids and the production of pro-inflammatory cytokines [interleukin-6 (IL-6), CXCL2, NLRP3, tumor necrosis factor-alpha (TNF-α) , and interleukin 1-beta (IL-1β)]. Gut microbes can also regulate cortisol release by affecting the activation state of the HPA. This in turn has an effect on microglia activation, cytokine release, and monocytes recruitment.

Bostanciklioğlu et al. found that gut metabolites can affect learning and memory through vagal afferent fibers that control the secretion of bioactive molecules (peptide YY, cholecystokinin, glucagon-like peptide-1) by enteroendocrine cells [78]. Therefore, AD pathology may result from a misregulated HPA axis and vagus nerve signaling induced by gut dysbiosis. In addition, dysbiosis of the gut microbiota can inhibit the expression of BDNF, a gene associated to neurogenesis and neuronal growth [72]. Dysbiosis induced by CRS translates into a decrease in the proportion of beneficial microorganisms (*Lactobacillus* and *Bifidobacterium*) and an increase in that of the more harmful ones (*Escherichia*, *Shigella*, *Proteus*, *Klebsiella*), leading to a decrease in the production of short chain fatty acids (SCFAs) [72, 79, 80]. SCFAs have a beneficial effect on the CNS and peripheral circulation and have been shown to play a key role in microbiota-gut-brain communication. SCFAs can interfere with various forms of Aβ peptides, effectively inhibiting aggregation of Aβ fibrils and reducing the accumulation of neurotoxic oligomers in the brain [81]. Dysbiosis of the gut microbiota therefore further decreases intestinal barrier integrity due to this reduction in SCFAs synthesis [73, 82]. SCFAs can also influence neuroinflammation by affecting microglial cell morphology and function as well as immune cells and immune modulators. Therefore, downregulation of SCFAs may potentially induce impairments in cognition, memory, and emotional response [83].

Recent investigations of microbial endocrinology also demonstrated that neuroactive molecules, such as neurotransmitters produced by gut microbes, can directly contribute to the crosstalk between the gut and the brain [84]. Acetylcholine, γ-aminobutyric acid (GABA), dopamine, and 5-hydroxytryptophan (5-HT), produced by gut microbes from the *Bifidobacterium* and *Lactobacillus* genera, among others, can influence nerve physiology. During CRS-induced dysbiosis, the secretion of these neurotransmitters is decreased, which is a predisposing factor to AD. In addition, gasotransmitters of microbial origin, including nitric oxide (NO), hydrogen sulfide (H_2_S), ammonia, and methane, also play crucial functions in neurophysiology, and may be participated in AD pathogenesis [85, 86]. For example, the elevation of NO increases BBB permeability. Furthermore, NO reacts with superoxide to form peroxynitrite, a potent oxidizing agent that can cause neurotoxicity. Oxidative stress and mitochondrial dysfunction, two well-characterized pathological features in AD, can also be induced by elevation of NO levels, leading to neuronal apoptosis. Moreover, oxidative stress can enhance Aβ production and deposition. Overproduction of H_2_S leads to decreased oxygen consumption of mitochondria and increased expression of pro-inflammatory factors such as IL-6 [87].

Based on the pieces of evidence discussed above, we may conclude that dysbiosis induced by CRS influences AD pathology in several ways. These include increased gut, vasculature, and BBB permeability; accelerated aging; increased amyloid burden; abnormal LPS secretion; misregulation of the HPA axis and vagus nerve signaling; neuroinflammation; oxidative stress; aberrant immune activity; alterations in the biodiversity and composition of the gut microbiota; downregulation of BDNF and SCFAs; decreased neurotransmitters secretion; and abnormal release of gasotransmitters (Fig. 5).

## 4. Social isolation stress

Social isolation stress (SIS) is a form of chronic stress that refers to a complete or almost complete lack of contact with conspecifics [88, 89]. Since humans are highly social, SIS can affect people of all ages and is known to be a trigger for emotional problems and cognitive dysfunction in adolescents [90]. SIS is also associated with an increased risk of death in the elderly. Robert et al. found that the incidence of AD in individuals affected by SIS was more than double that of the control group [91]. The recent Lancet Commission on Dementia Prevention, Intervention, and Care estimated that if the risk of SIS in later life was eliminated, the prevalence of dementia would be reduced by 4%. The impact would be greater than that estimated for reducing physical inactivity in later life (2%) or hypertension in midlife (2%).

Positron emission tomography (PET) imaging has shown that Aβ protein load is significantly correlated with increased loneliness [92]. Changes in some pathological markers of AD, such as increased amyloid beta plaques and neurofibrillary tangles, are not always equivalent to the degree of cognitive decline or clinical dementia. The closest neurobiological association with cognitive decline in AD is synaptic degeneration and/or loss. Therefore, many technologies that can directly measure biomarkers of synapse loss or damage have already been adopted in clinical settings. These include PET ligands that label synapses in vivo and biomarkers that detect synaptic degeneration in the cerebrospinal fluid [93-95]. The emergence of these technologies provided new evidence indicating that SIS affects the onset of AD by inducing increased synaptic degeneration and/or loss [96].

It is well established that various SIS models can induce AD-type pathological features (Table 2). APP/PS1 mice showed normal hippocampal long-term potentiation (LTP) and situational fear conditioning at the age of 3 months, indicating that they had no defects in learning and memory [97]. However, when APP/PS1 mice were raised in social isolation, cognitive impairment was already evident at 3 months of age and was accompanied by a massive increase in Aβ. This latter effect was due to a significant increase in the activity of β- and γ-secretase, resulting in excessive production of Aβ_40_ and Aβ_42_ in the hippocampus [98]. In addition, the researchers also found that SIS increased calpain activity and the p25/p35 ratio, while reducing membrane-associated p35. The main reason for this was that the large amount of Aβ promoted calcium influx and calpain activation. Calpain-mediated proteolysis releases p25 from the N-terminus of p35, and an increased p25/p35 ratio promotes tau phosphorylation and induces neuronal death [99]. Furthermore, membrane-associated p35 interacts with the AMPA receptor subunit GluR1 and α-CamKII to form the p35-GluR1-CamKII complex. SIS reduces the interaction of p35-GluR1-CaMKII. Since the p35-GluR1-CaMKII complex is important for synaptic plasticity, learning, and memory, this decrease in the formation of the complex resulting from SIS leads to memory and cognitive impairment in APP/PS1 mice [100, 101]. Cao et al. raised APP/PS1 transgenic AD mice singly for 8 weeks at the age of 1 month. They found SIS increased hippocampal cell apoptosis, synaptic protein loss, glial activation, and triggered inflammatory responses by increasing the expression of IL-1β, IL-6, and TNF-α [102].

Huang and colleagues housed seventeen-month-old APP/PS1 mice in isolation for 3 months and found that this exacerbated hippocampal atrophy, increased the accumulation of hippocampal Aβ plaques, and induced cognitive dysfunction. Expression of γ-secretase was increased, and that of neprilysin (NEP) was decreased. Synapse and myelin loss, as well as glial neuroinflammatory reactions, were exacerbated [88]. NEP and insulin-degrading enzymes (IDE) are two major Aβ-degrading enzymes that play a vital role in maintaining Aβ homeostasis in the brain via Aβ degradation. NEP expression is negatively correlated with Aβ accumulation and cognitive impairment severity, while the expression levels of IDE do not seem to be correlated with these two events. In addition, similarly to what has been noticed for CRS, the large amount of Aβ induced by SIS can promote intracellular calcium ion overload and tau protein hyperphosphorylation, disrupt mitochondrial energy metabolism, aggravate oxidative stress, and activate neuronal apoptosis and other pathways that affect the normal structure and function of the hippocampus negatively [103].

In another APP transgenic mouse model (Tg2576), the subjects developed normally until 9 months of age, and almost no β-amyloid deposits are detectable in the brain [104]. After these mice were individually housed in special cages one-third the size of a standard mouse cage from weaning to 6 months, a large number of senile plaques formed by the deposition of Aβ_42 were_ observed in the brain, resulting in impaired the ability to generate new cells in the dentate gyrus of the hippocampus [105]. Neurogenesis of the hippocampal dentate gyrus is thought to be related to learning and memory [106]. Increased Aβ deposition can damage neurons by disrupting intracellular calcium ion homeostasis, inducing oxidative stress, and causing massive release of glutamate [107]. Studies in rats have found that SIS during 6 consecutive weeks can induce tau hyperphosphorylation and deficits in learning and spatial memory in middle-aged rats [89]. Research into the underlying mechanism showed that SIS inhibited the phosphorylation of GSK3β at Ser9, resulting in an increase in GSK3β activity. Since GSK3 kinase plays an essential role in regulating tau phosphorylation, this in turn led to tau hyperphosphorylation and deficits in spatial memory. In addition, the BDNF/PI3K/Akt/GSK3β signaling pathway plays important roles in synapse formation, neuronal differentiation and survival, and regulation of synaptic structure and function [108]. Gong et al. found that SIS reduced the expression of BDNF, serine 473-phosphorylated Akt, and serine 9-GSK3β [109]. Ali et al. found that SIS increased β-secretase, Aβ protein, Tyr-216-GSK3β, phosphorylated tau, malondialdehyde, IL-1β, and TNF-α gene expression levels [110].

**Table 2:** Rodent studies on impact of social isolation stress (SIS) and chronic noise exposure (CNE) on AD-related markers.

Stress paradigm	Procedure	Animal model	Relevance to AD	Ref.
**SIS**	Mice were singly housed for 3 months.	APP/PS1 transgenic mouse	Cognitive dysfunction and Aβ plaque accumulation ↑ Exacerbated hippocampal atrophy	88
	Isolated rats were housed in cages measuring 38× 22×20 cm, while group rats were maintained in cages measuring 48×30×20 cm, for 6 weeks.	Sprague-Dawley rat	p-tau ↑ Spatial memory deficit	89
	Mice were individually housed (one mice per cage) for a period of about 7 weeks.	APP/PS1 transgenic mouse	Aβ_40_ and Aβ_42_ ↑ Memory impairment	98
	Mice were individually housed (one mice per cage) for a period of about 8 weeks.	APP/PS1 transgenic mouse	p-tau, Aβ_40_ and Aβ_42_ ↑ Accelerated cognitive and memory deficits	100
	Mice were individually housed in cages one third the size of a standard cage and placed in a separate room from weaning until 6 months of age.	Tg2576 transgenic mouse	Aβ_42_ and β-amyloid plaque deposition ↑ Contextual memory ↓	105
	Isolated rats were housed individually in cages, while the same size of the cages housed 4 control animals, for 5 weeks.	Wistar rat	Aβ and p-tau ↑ Spatial learning and memory ↓	110
**CNE**	Exposed to 75 dB noise 8 h/d either for the light-cycle group or the dark-cycle group, for 30 days.	C57BL/6NJ mouse	Impairments in learning and memory Hippocampal volume ↓	114
	Emitted an intermittent 3000Hz frequency sound of 90 dB for 1 sec in the intervals of 15 sec during 24 h.	C57BL/6 mouse	Learning and memory performance ↓	115
	80 dB, frequency was from 10 to 10,000 Hz, 2h/d, for 6 weeks.	Kunming mouse	p-tau ↑ Impaired the learning and memory ability	117
	100 dB white noise, 4 h/d, for 14 days.	Wistar rat	p-tau ↑ Formation of pathological neurofibrillary tangle	120
	Exposed to noise for 4 h every day up to 14 days at 110 dB of noise level.	C57BL/6 mouse	Spatial memory deficits	126

## 5. Chronic noise stress

Noise stress is harmful, particularly to the CNS. According to previous reports, chronic noise exposure (CNE) can induce cognitive impairment and is also a predisposing factor for AD pathogenesis [111-113]. There is a compelling body of research on the effect of CNE on pathological features and mechanisms associated with AD (Table 2) which will be discussed below.

CNE can promote the secretion of CRF and glucocorticoids by activating the HPA axis and the CRF pathway, thereby promoting tau phosphorylation and other AD-related pathologies [114-116]. Hyper-phosphorylation of tau reminiscent of what is observed in AD has been found in the brains of rats exposed to long-term noise. In this rat model, the increase in tau phosphorylation differs between chronic and acute stress, but both can cause cognitive impairment [117, 118]. In the chronic stress model, the phosphorylation of tau is permanently increased, whereas in the acute stress model, tau begins to dephosphorylate 24 h after the stressor is removed [119]. The expression of PP2A in the rat model of CNE is increased, which is the opposite of what has been observed in the AD brain. It has been postulated that when the level of PP2A increases, the increase in GSK3β activity is responsible for the tau phosphorylation, thereby counteracting the dephosphorylation effect of PP2A [120]. Other studies have found Aβ production and abnormal phosphorylation of tau were evident in the brains of Kunming mice and SAMP8 after CNE [117, 121].

CNE can induce neuronal damage, particularly in the hippocampus, ultimately causing neuronal loss and memory impairments [114]. The hyperphosphorylation and aggregation of tau caused by CNE results in neurofibrillary tangles, and the massive production and deposition of Aβ can cause disorders of synaptic function and apoptosis of nerve cells, ultimately also inducing learning and memory impairments. The hyperphosphorylation of the tau is mediated by the GluN2B subunit of NMDARs. Once this signaling pathway is overactivated, the kinases GSK3β and CDK5 associated with tau phosphorylation are activated through the GluN2B-Fyn signaling pathway. GluN2B can also directly inhibit the activity of PP2A [122]. Other studies have reached similar conclusions, namely, CNE-induced disruption of the NMDAR signaling pathway ultimately leads to high phosphorylation of tau. Use of the NMDAR antagonist MK-801 can reverse the activation of GSK3β and the increase in tau protein phosphorylation induced by noise stress [123]. In addition, CNE can increase the level of glutamate in the brain and thus promotes the influx of Ca^2+^, which triggers ROS production and inhibits LTP [124].

Other researchers have determined that oxidative stress can promote the generation and deposition of Aβ as well as tau phosphorylation. Oxidative stress is increased in response to noise stress and could therefore mediate the occurrence of AD-type pathological features [125, 126]. The main effects of oxidative stress are increased APP expression, decreased α-secretase activity, and increased expression and activation of β- and γ-secretase [127].

## 6. Chronic unpredictable mild stress

Chronic unpredictable mild stress (CUMS), also called chronic variable stress, refers to an inconsistency in the exposure to stress or to the use of multiple different forms of stress in a single stress model to achieve unpredictable effects. As the stress-inducing procedure varies among different studies, it is difficult to compare the results and draw any general conclusion (Table 3). For example, in a study by Han et al., the results of their CUMS procedure did not show any increase in Aβ_40_ and Aβ_42_ in the mouse hippocampus. In another study involving APP/PS1 mice, CUMS was introduced under the same conditions, and in this instance, it not only promoted the expression of Aβ_40_ and Aβ_42_, but also induced neuronal injury and cognitive impairment [128]. Consistent with Han’s report, Bing et al. found that CUMS induced Aβ deposition and severe impairment of cognitive behavior in APP/PS1 mice for 4 weeks, but they observed no significant effect on wild type C57 mice. The authors believe that the main reason behind these contradictory results is that six-month-old C57 mice have better tolerance to CUMS because they are at the peak of brain function development. In addition, these results are clearly dependent on the physical conditions of the mice and the skills of the experimenters [129]. Hossein et al. used another model of CUMS and found that it significantly increased Aβ levels in the hippocampus of adult male rats [130], but the effect on tau phosphorylation was not reported. We previously exposed ten-week-old C57 mice to CRS for 4 weeks and detected tau hyperphosphorylation in the hippocampus and prefrontal cortex [37]. In addition, Carroll et al. found that exposure to CRS rather than CUMS for one month exacerbated Aβ levels in Tg2576 transgenic mice [7].

The results obtained in studies that adopted the unpredictable stress model are diverse. It has been reported in the literature that after 4 consecutive weeks of CUMS in wild type rats, tau is abnormally phosphorylated in the hippocampus and prefrontal cortex, and behavioral tests shown a decline in learning and memory ability [131]. Another study reported that CUMS can induce the production of Aβ_42_ in the hippocampal CA1 region of rats [132]. Research by Peay et al. found that CUMS only damaged the spatial memory of male rats but had no significant effect on female rats [133]. Studies have confirmed that abnormal expression or activation of Fyn is closely related to tau phosphorylation, amyloid accumulation, and cognitive decline in patients with AD [134-136] and blocking the abnormal activation of Fyn can protect neurons from Aβ toxicity [137]. Lopes et al. utilized another model of CUMS that could induce tau phosphorylation, neuronal atrophy, dendritic spine shortening, and learning and memory disorders in 4- to 6-month-old wild-type mice and found that CUMS upregulated Fyn expression in the hippocampus [138]. Upregulated Fyn interacts with PSD-95 and GluN2B to form a GluN2B/PSD-95/Fyn complex, which regulates the activation of glutamate NMDARs. The activation of NMDARs in turn activates two key tau protein kinases, GSK3β and CDK5. Furthermore, Fyn also plays an important role in mediating tau-induced neuropathology [139].

Four-month-old Tg2576 transgenic mice were subjected to CUMS for 7 weeks. Almost no AD-type pathological features were detected in the brains of the control group, and their behaviors were normal, whereas the CUMS-exposed group showed Aβ deposition and tau phosphorylation in the brain. Cognitive impairment was also demonstrated in this study using the water maze test. The proposed mechanism was an increase in the activity of β-secretase and an inhibition in the expression of Ser9-GSK3β [140], which is similar to the mechanism underlying the pathological changes in response to noise stress.

**Table 3:** Rodent studies on impact of chronic unpredictable mild stress (CUMS) on AD-related markers.

Stress paradigm	Procedure	Animal model	Relevance to AD	Ref.
**CUMS**	The stressors included: (1) long swim for 20 min in a 30 °C water bath, (2) cold swim for 2.5 min in a 15 °C water bath, (3) restraint for 15 min in a 50 ml conical tube, (4) housed in isolation for 24 h, (5) housed with soiled bedding for 24 h, and (6) housed under lights-on conditions for 24 h. One stressor per day for 6 d/week, and for 4 weeks.	Tg2576 transgenic mouse	No changes on Aβ_40_, Aβ_42_ and p-tau Displayed no spatial memory impairment Displayed impairment on both context and cued fear conditioning	7
	The stressors included: (1) food deprivation for 24 h, (2) water deprivation for 24 h, (3) overnight illumination, (4) removal sawdust for 24 h, (5) soiled cage for 24 h, (6) forced swimming at 8 °C for 6 min, (7) tail nipping, and (8) physically restraint for 2 h. Stressors 2-3 times a day for 4 weeks.	C57BL/6 mouse	No changes on Aβ_40_, Aβ_42_ and senile plaque deposition Cognitive deficiency	128
		APP/PS1 transgenic mouse	Aβ_40_, Aβ_42_ and senile plaque deposition ↑ Significantly cognitive deficiency	
	The stressors included: (1) bending the cage at 45° for 24 h, (2) 24 h contact with wet bedding, (3) swimming in 4 °C water for 5 min, (4) swimming in 45 °C water for 5 min, (5) overnight illumination, (6)12 h of food deprivation, and (7)12 h of water deprivation. One stressor per day for 4 weeks.	Wistar rat	Aβ ↑ Impairment of learning and memory	130
	Application of one of the following stressors, daily: hypertonic saline, overcrowding for 1 h, placement in a confined environment (30 min), or placement on a vibrating/rocking platform (1 h); for 1 month.	Wistar rat	p-tau ↑ Deficits in learning and memory	131
	Application of one of the following stressors, daily: 24-h food deprivation, 24-h water deprivation, 5-min cold swimming (6 °C), 1-min tail pinch, physically restraint for 2 h, exposure to rat odor for 1 h and overnight illumination; for 4 weeks.	Wistar rat	Soluble and insoluble Aβ_42_ ↑ Deficits in learning and memory	132
	Animals were exposed to one of the following stressors once a day for 1 hour: restraint, vibrating platform, overcrowding, or a hot air stream on consecutive days; for 6 weeks.	C57BL/6J mouse	p-tau ↑ Impairments in spatial learning and memory	138
	Application of 2-3 following stressors in any 24 h period: Low intensity stroboscopic illumination (8 h), intermittent bell ringing (10 db, 1/10 s), or white noise (4 h), 45° cage tilt (8 h), damp bedding (6 h), rat odor (8 h), darkness during the day (3 h), transfer of cages to another room (4 h), placement of a novel object in the cage (3 h), overnight water and food deprivation, illumination and removal of nesting material (12 h), and swimming in cold water (18 °C, 5 min); for 6 weeks.	Tg2576 transgenic mice	Aβ_40_, Aβ_42_ and senile plaque deposition ↑ p-tau ↑ Impairment of learning and memory	140

## 7. Conclusion

We are inevitably affected by stressful events. It is of great significance to investigate the pathological features and mechanisms responsible for AD in response to chronic stress. A reliable animal stress model is essential for this purpose. Although not all stress animal models can fully replicate the AD-type pathological features, tau hyperphosphorylation, Aβ overproduction and deposition, and learning, memory, and cognitive dysfunction can be induced in most of them (Table 1, Table 2, Table 3). HPA axis dysfunction, abnormalities in the CRF system and glutamate systems, neuro-inflammation, aberrant immune activity, dysbiosis of the gut microbiota, downregulation of neurotrophic factors, synaptic degeneration, and changes in the activity and expression of GSK3β, CDK5, and PP2A in response to chronic stress are all of great significance in the pathogenesis of AD. This line of research provides a basis for the development of more effective prevention and treatments strategies aimed at improving the quality of life of affected individuals.
